# Biochemical characterization of naturally occurring mutations in SARS‐CoV‐2 RNA‐dependent RNA polymerase

**DOI:** 10.1002/pro.5103

**Published:** 2024-08-15

**Authors:** Matěj Danda, Anna Klimešová, Klára Kušková, Alžběta Dostálková, Aneta Pagáčová, Jan Prchal, Marina Kapisheva, Tomáš Ruml, Michaela Rumlová

**Affiliations:** ^1^ Department of Biotechnology University of Chemistry and Technology Prague Czech Republic; ^2^ Department of Biochemistry and Microbiology University of Chemistry and Technology Prague Czech Republic

**Keywords:** mutations, phenotypic effect, RNA‐dependent RNA polymerase (RdRp), SARS‐CoV‐2

## Abstract

Since the emergence of SARS‐CoV‐2, mutations in all subunits of the RNA‐dependent RNA polymerase (RdRp) of the virus have been repeatedly reported. Although RdRp represents a primary target for antiviral drugs, experimental studies exploring the phenotypic effect of these mutations have been limited. This study focuses on the phenotypic effects of substitutions in the three RdRp subunits: nsp7, nsp8, and nsp12, selected based on their occurrence rate and potential impact. We employed nano‐differential scanning fluorimetry and microscale thermophoresis to examine the impact of these mutations on protein stability and RdRp complex assembly. We observed diverse impacts; notably, a single mutation in nsp8 significantly increased its stability as evidenced by a 13°C increase in melting temperature, whereas certain mutations in nsp7 and nsp8 reduced their binding affinity to nsp12 during RdRp complex formation. Using a fluorometric enzymatic assay, we assessed the overall effect on RNA polymerase activity. We found that most of the examined mutations altered the polymerase activity, often as a direct result of changes in stability or affinity to the other components of the RdRp complex. Intriguingly, a combination of nsp8 A21V and nsp12 P323L mutations resulted in a 50% increase in polymerase activity. To our knowledge, this is the first biochemical study to demonstrate the impact of amino acid mutations across all components constituting the RdRp complex in emerging SARS‐CoV‐2 subvariants.

## 1 INTRODUCTION

The coronavirus disease 2019 (COVID‐19) emerged in December 2019 and shortly after escalated into a global pandemic. As of December 24, 2023, there have been 773,119,173 reported cases with 6,990,067 fatalities (WHO, 2023a). Although the World Health Organization has declared that COVID‐19 is no longer a public health emergency of international concern, it continues to present a severe health threat, particularly to vulnerable groups (WHO, 2023b). The causative agent of COVID‐19 is severe acute respiratory syndrome coronavirus 2 (SARS‐CoV‐2). Typically, coronaviruses cause mild to moderate respiratory illnesses. However, specific strains within the *Betacoronavirus* genus have been associated with severe outbreaks, namely SARS‐CoV, which led to the severe acute respiratory syndrome (SARS) outbreak in 2003, and MERS‐CoV, known for the middle east respiratory syndrome (MERS) (Song et al., 2019).

Betacoronaviruses are enveloped viruses with a positive‐sense single‐stranded RNA genome of ~30 kilobases (kb). This categorizes them among the largest known RNA viruses (Gorbalenya et al., 2006). Their genome encodes four structural proteins, and multiple nonstructural and accessory proteins. A major part of the genome is represented by ORF1a and ORF1ab, which are translated into two large polyproteins pp1a and pp1ab. Upon translation, the pp1a and pp1ab polyproteins undergo cleavage, mediated by a papain‐like protease, resulting in the production of 10 and 16 nonstructural proteins (nsps), respectively. The main role of these nonstructural proteins is to mediate the transcription and replication of viral RNA through various mechanisms. Out of all the 16 nsps, nine (nsp7–nsp10; nsp12–nsp16) assemble to form the coronaviral replication‐transcription complex (RTC), which is directly responsible for RNA replication (Snijder et al., 2016; Weiss & Leibowitz, 2011; Wu et al., 2020).

Amidst other replication factors in the RTC, nsp12, a canonical RNA‐dependent RNA polymerase (RdRp), possesses pivotal enzymatic activity. Structurally, nsp12 adopts a typical “right‐hand” architecture with subdomains corresponding to the thumb, palm, and fingers. These motifs, conserved among the RNA viruses (McDonald, 2013), collectively form the catalytic site of the polymerase. Furthermore, nsp12 includes a nidovirus RdRp‐associated nucleotidyl transferase (NiRAN) domain participating in the capping process (Kirchdoerfer & Ward, 2019; Walker et al., 2021). On its own, nsp12 exhibits only minimal RdRp activity. It gains most of its catalytic activity upon binding to accessory subunits nsp7 and nsp8, forming minimal complex capable of mediating RNA replication, nsp7:nsp8:nsp12 (Subissi et al., 2014). This RdRp complex contains a single nsp7 and two nsp8 subunits; nsp8a directly interacts with nsp12, while nsp8b is bound via nsp7. While nsp7 is a small protein consisting of three *α*‐helices, each nsp8 subunit has a “golf club”‐like shape, with a C‐terminal head domain and a rod‐like N‐terminal extension that stabilizes both the newly synthesized RNA and template duplex (Figure 2a) (Hillen et al., 2020; Subissi et al., 2014). The RdRp of SARS‐CoV‐2, like other RNA viruses, operates with low intrinsic fidelity (Yin et al., 2023). To replicate its 30 kb long genomic RNA effectively and avoid lethal mutagenesis, coronaviruses developed an elaborate mechanism utilizing a viral 3′–5′ exoribonuclease (nsp14) as an independent proofreading factor (Gorbalenya et al., 2006; Ogando et al., 2020).

Mutations are the driving force behind the evolution and adaptation of RNA viruses. The evolution of mutant variants is closely linked to the pathogenesis and diversity in the viral population (Stern & Andino, 2016). SARS‐CoV‐2 thus represents a perfect example of a rapidly evolving RNA virus. Five variants of concern (VOCs), designated Alpha, Beta, Gamma, Delta, and Omicron, have been described since the first appearance of SARS‐CoV‐2 in December 2019. These variants were primarily classified based on mutations in the spike protein (S). Due to its role in immune response, target recognition, and cellular entry, the mutations in S proteins are directly involved in virulence and the infectious cycle. However, despite occurring regularly, mutations in nonstructural and accessory proteins have been studied to a much lower extent (Bakhshandeh et al., 2021; Carabelli et al., 2023; Magazine et al., 2022).

Previous research on SARS‐CoV‐2 RdRp mutations has been focused mainly on nsp12 (Kim et al., 2023; Miropolskaya et al., 2023), although Subissi et al. (2014) demonstrated that mutations in accessory subunits, nsp7 and nsp8, can significantly influence virus fitness. Some of the polymorphisms of both nsp7 and nsp8 have been explored in silico, to predict their potential impact on the RdRp complex (Reshamwala et al., 2021; Senthilazhagan et al., 2023). Despite the key role of RdRp in the virus life cycle, its significance in the development of drugs against COVID‐19 (Ng et al., 2022; Xu, Chen, et al., 2022) and recognizing the potential for amino acid changes in viral enzymes to induce drug resistance (Nakamoto et al., 2014; Piret & Boivin, 2011; White et al., 2002), our knowledge of polymorphisms in the RdRp proteins remains rather limited.

To address this, we conducted a comprehensive characterization of representative naturally occurring mutations in nsp7, nsp8, and nsp12. We examined the biophysical properties of these proteins and assessed the overall enzymatic activity of the RdRp complex variants. Our results elucidate how natural mutations impact the entire RdRp complex and are thus invaluable for future drug research.

## 2 RESULTS

### 2.1 Selection of representative mutations in nsp7, nsp8, and nsp12

**FIGURE 1:**
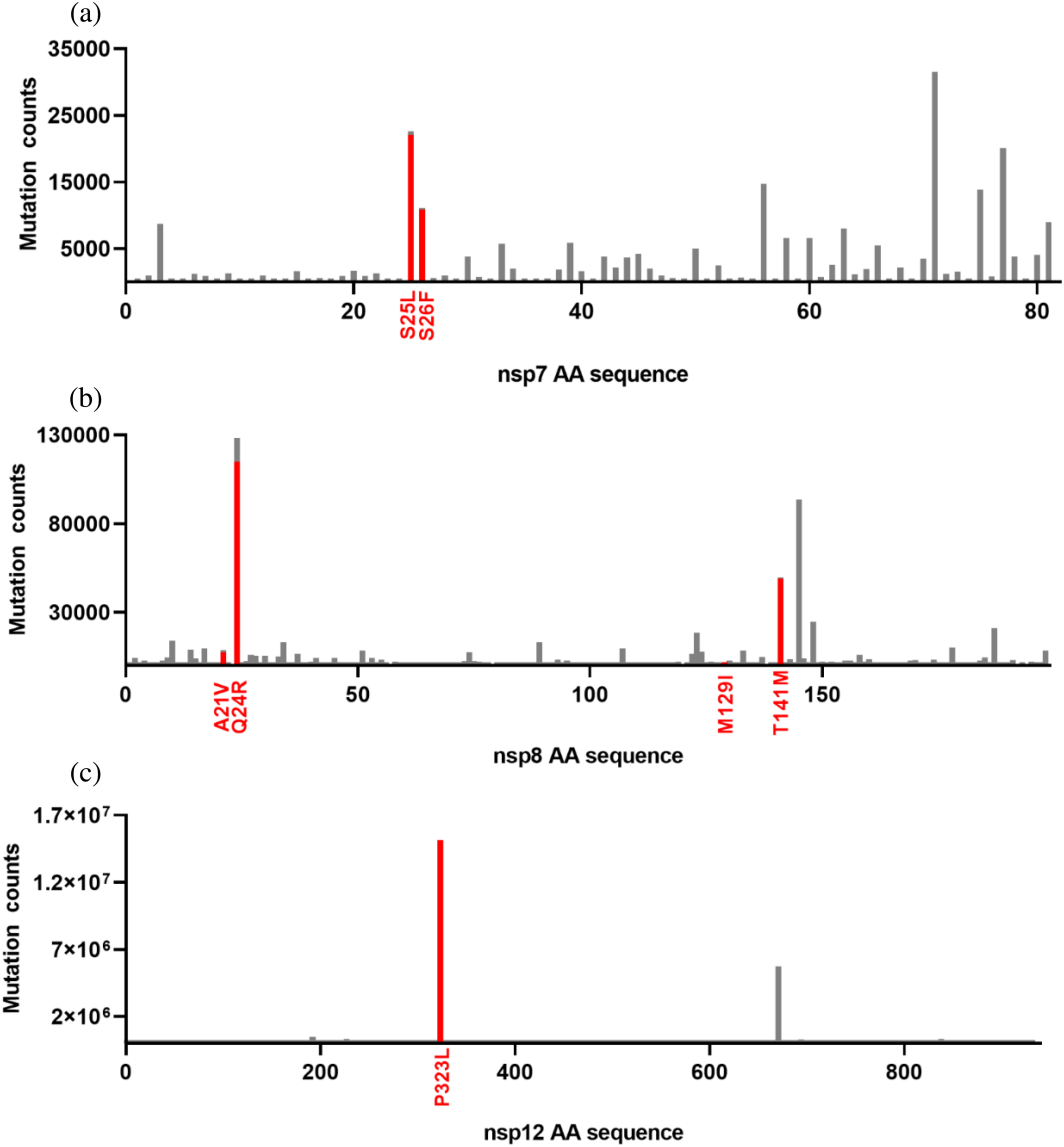
Frequency of amino acid substitutions within the RdRp complex protein subunits. The graphs illustrate the sums of all different mutations at each position within the amino acid sequences of the proteins comprising RdRp complex. The selected amino acid substitutions are depicted in red: (a) amino acid exchanges in the nsp7 sequence, (b) amino acid substitutions within the nsp8 sequences and (c) identified amino acid substitutions in nsp12. The source data were retrieved from (covidcg.org) over the period from January 2020 to June 30, 2022. The entire dataset subjected to analysis comprised 15,243,203 sequences.

**FIGURE 2:**
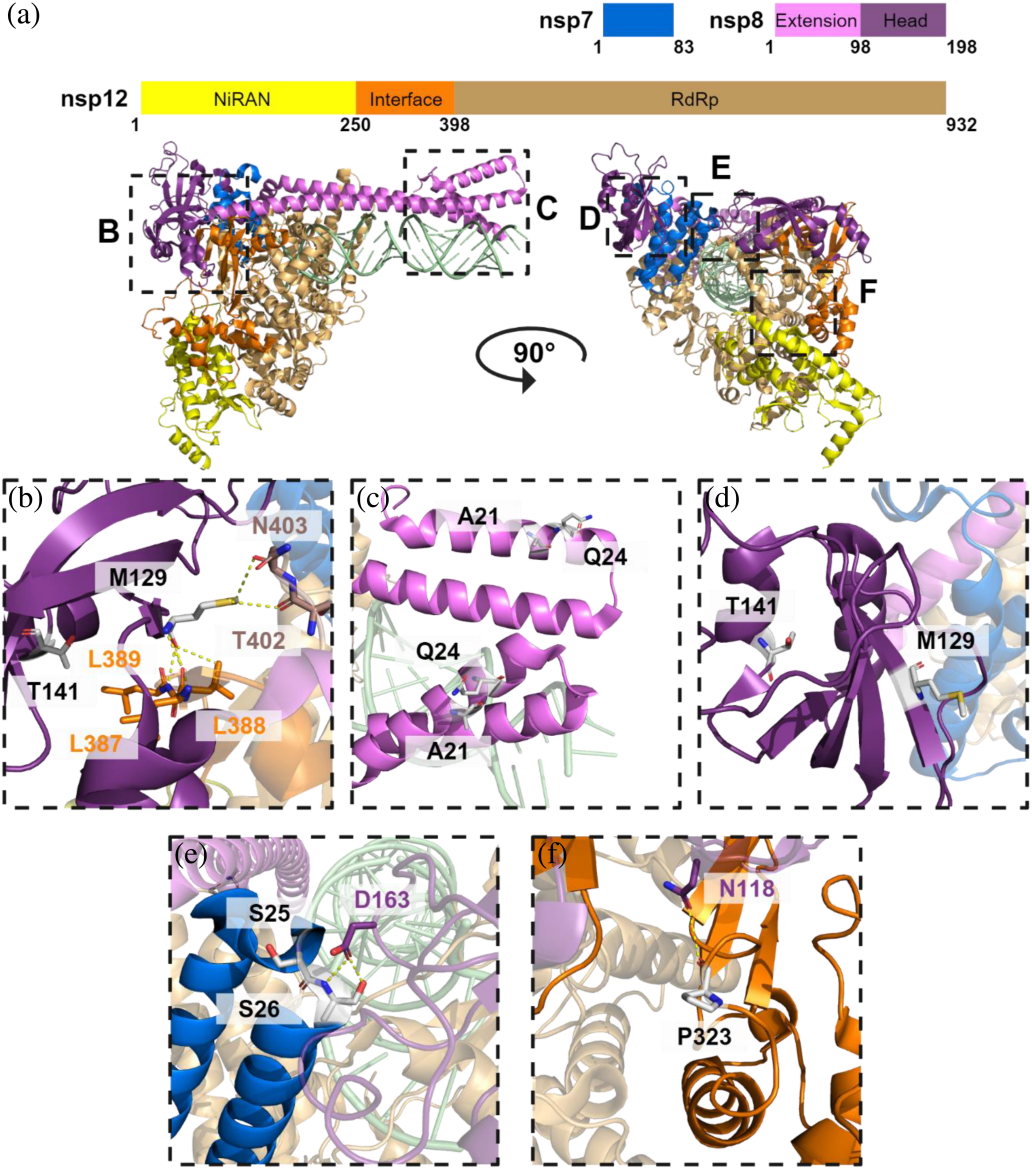
Schematic representation of SARS‐CoV‐2 RdRp complex and selected amino acid residues in RdRp protein subunits. (a) A model structure (PDB id: 6yyt (Hillen et al., 2020)) and domain description of the proteins comprising the SARS‐CoV‐2 minimal RdRp complex: A single nsp7 (blue) and two nsp8 subunits are bound to nsp12; nsp8a directly and nsp8b through nsp7. Nsp8 consists of two subdomains—a head domain (magenta) and a rod‐like helical extension (pink) that stabilizes the nascent RNA duplex (green). The multidomain nsp12 is composed of the polymerase (wheat), interface (orange), and NiRAN (yellow) domains. The localization of amino acid residues selected for this study are visualized in close‐up views at panels b, c, d, e, and f: (b) view of the nsp8a interaction with nsp12 interface and RdRp domains, (c) close‐up view of the nsp8 extension domains, (d) selected amino acid residues in nsp8b, (e) nsp7 loop interacting with nsp8b and (f) the P323 residue in nsp12 interface domain and its interaction with nsp8b.

To select the representative mutations in RdRp subunits, we analyzed the natural amino acid substitutions occurring in nsp7, nsp8, and nsp12 over the period from January 2020 to June 30, 2022, covering all five SARS‐CoV‐2 VOCs (Figure 1). This search was based on the data in the public database (covidcg.org), encompassing a total of 15,243,203 sequences. Despite their general conservation, as indicated by data from the public database, numerous natural substitutions in RdRp accessory subunits, nsp7 and nsp8, as well as in nsp12, were observed during the pandemic. Using an experimental RdRp structure PDB ID: 6yyt (Hillen et al., 2020) (Figure 2a), we focused on amino acid residues at the interfaces that are involved in protein–protein interactions within the RdRp complex. We also considered the possibility of the mutations significantly altering the chemical properties of the respective residue.

We observed that the most frequent mutations in the nsp7 sequence were primarily located in the N‐terminal region spanning residues 24–26, and in the C‐terminal region, specifically involving residues 56, 71, and 77 (Figure 1a). Due to the limited structural characterization of this nsp7 region (Gao et al., 2020; Hillen et al., 2020; Wilamowski et al., 2021; Zhang et al., 2021), we focused on the residues close to the loop connecting helices 1 and 2: S24, S25, and S26 (Figure 2e) and situated at the top of the RdRp complex directly interacting with D163 in nsp8a. Two relatively frequent amino acid substitutions—S25L and S26F—have been identified in this region. Both of these replace the serine residue with a hydrophobic one, potentially influencing the interaction dynamic of the loop.

In nsp8, Q24 is the most frequently altered amino acid residue (Figure 1b). It is located in the short *α*‐helix at the end of the extension domain (Figure 2c). Interestingly, in existing SARS‐CoV‐2 variants, this amino acid residue is most commonly replaced by positively charged amino acids, especially arginine. Apart from this Q24R substitution, we also selected a more subtle, yet frequent substitution, A21V (Figure 2c). The head domain of nsp8 exhibits several threonine mutations, including T141, T145, or T148 (Figure 1b). Among these, we particularly focused on T141M, which is the second most prevalent mutation within this subdomain with its position in close proximity to nsp12 in nsp8a (Figure 2b). Another area of interest was the more conserved region of the nsp8 head subdomain where the M129I substitution occurs. This substitution introduces a highly hydrophobic residue at the nsp8a:nsp12 interface. Moreover, M129 directly interacts with several residues in nsp12 (Figure 2b). Neither of the two selected residues contributes to nsp8b:nsp12 interaction (Figure 2d). Intriguingly, the M129I substitution in nsp8 was exclusively identified in sequence variants lacking the currently prevailing P323L substitution in nsp12, as reported by June 30, 2022, comprising 99.18% of all analyzed nsp12 sequences (Figure 1c).

The P323 residue is located directly at the interface between nsp12 and nsp8a (Figure 2f), making it a critical site for examining interactions within the RdRp complex. The impact of this substitution on RNA polymerase activity and RdRp complex formation has been previously studied (Kim et al., 2023). We included the P323L substitution not only to assess its direct impact on the formation and stability of the RdRp complex but also to evaluate whether mutations in accessory subunits nsp7 and nsp8 can influence the RdRp to a similar extent as mutations in the main enzymatic unit nsp12.

### 2.2 Impact of selected mutations on protein and RdRp complex stability

**FIGURE 3:**
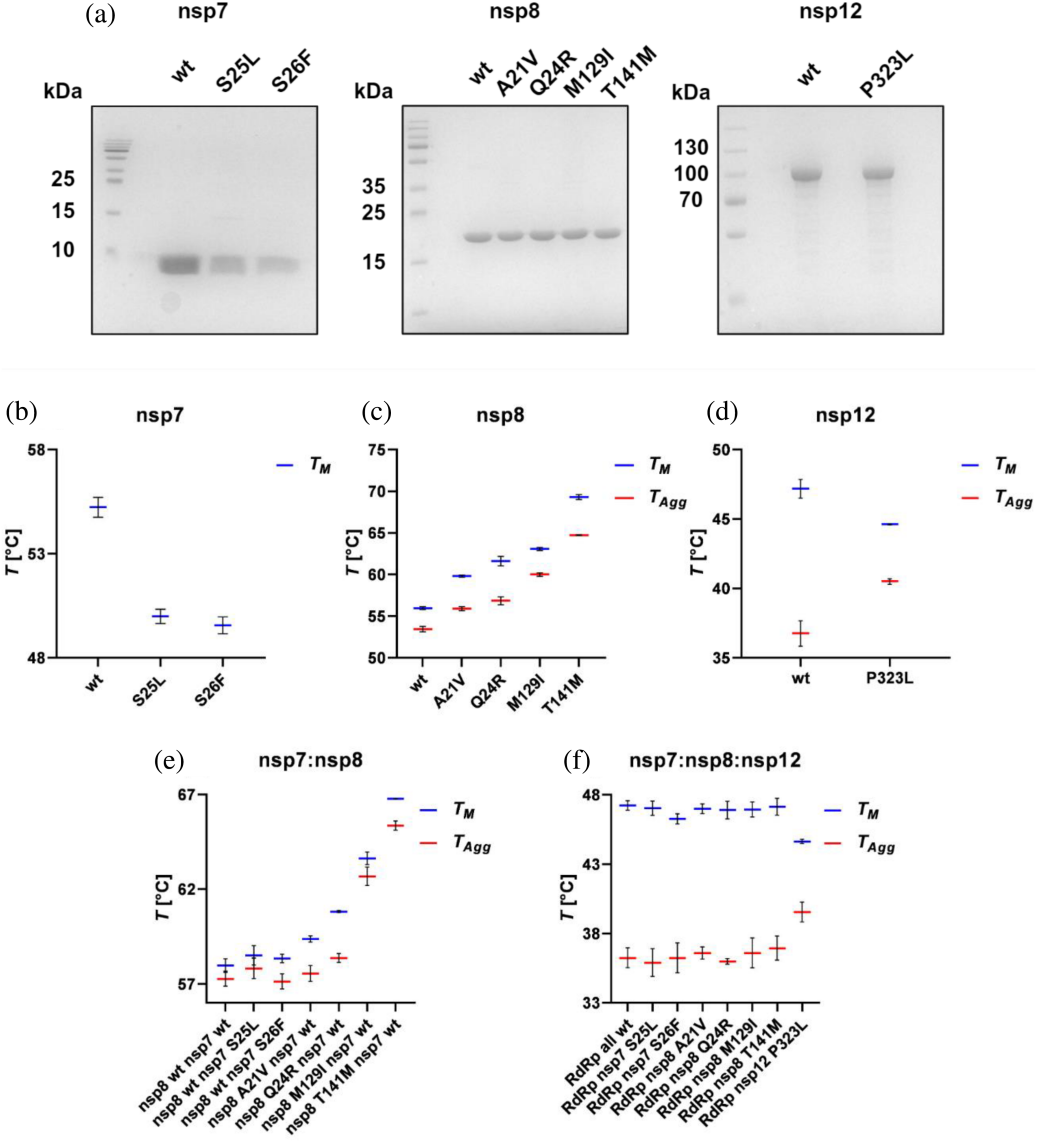
Assessment of protein stability of individual RdRp proteins and their complexes. (a) SDS‐PAGE analysis of purified nsp7, nsp8, and nsp12 variants. The thermal stability (TS) of individual RdRp proteins is shown in panels b, c, d: (b) TS of S25L and S26F of nsp7 mutants, (c) TS of nsp8 mutants, (d) TS of nsp12 wt and P323L. (e) TS of heterodimeric nsp7‐nsp8 complexes formed by wt and indicated nsp7 and nsp8 mutants was assessed. (f) TS of nsp12 mixtures with nsp7 and nsp8 variants in a 1:1:2 ratio (nsp12:nsp7:nsp8). The tested complexes were formed by mixing two wt components with one mutant component, for example, the RdRp nsp7 S25L complex was formed by wt nsp12, wt nsp8, and S25L variant of nsp7. The melting temperature (*T*_*M*_), shown in blue, determined using nanoDSF is represented by the inflection point in the 330/350 nm ratio curve. The turbidimetrically assessed aggregation temperature (*T*_*Agg*_), shown in red, represents the initial temperature at which the aggregation starts. The displayed results represent data from three independent measurements for each sample processed with the Panta analysis software.

To assess the impact of the selected amino acid substitutions on the stability and polymerase activity of the RdRp, we introduced these mutations into the respective expression vectors. Subsequently, we produced and purified all proteins of interest. The wild‐type (wt) nsp7 and nsp8 along with their mutant variants; nsp7 S25L and S26F and nsp8 A21V, Q24R, M129I, and T141M, were produced in *Escherichia coli*. Although the bacterial expression of nsp12 has been established for various purposes (Madru et al., 2021; Malone et al., 2023), it was also reported to potentially yield inactive or misfolded proteins, insufficient for functional studies (Wang et al., 2021). Therefore, we produced both the wt and P323L variants of nsp12 in the *sf9* insect cell line. The mutagenesis of nsp8 and nsp12 did not impact the established purification protocol for wt proteins, nor did it significantly affect the yield. However, the purification of nsp7 variants with introduced hydrophobic residues required optimization especially due to their decreased solubility. We enhanced solubility for S25L and S26F variants by adding a mild detergent to the lysis buffer, though the yield was lower in comparison to the wt (Figure 3a). Moreover, both mutant variants of nsp7 precipitated at higher concentrations.

Subsequently, the stability of individual proteins and their complexes was assessed. Melting temperatures (*T*_*M*_) and the temperatures of the aggregation onset (*T*_*agg*_) were determined using a thermal unfolding assay employing nano‐differential scanning fluorimetry (nanoDSF) and back‐reflection turbidimetric measurement, respectively, for each mutant. In nsp7 protein, the substitution of serine at positions 25 and 26 with either leucine or phenylalanine led to its destabilization, as evidenced by a decrease of *T*_*M*_ by ~4.5°C for both S25L and S26F compared to wt (Figure 3b, blue lines). No increase in turbidity was observed during the measurements, suggesting that none of the nsp7 variants aggregate as a result of thermal denaturation. In contrast, all analyzed amino acid substitutions in nsp8 stabilized the protein (Figure 3c, blue lines). Moreover, *T*_*agg*_ (Figure 3c, red lines) was similarly affected as *T*_*M*_, confirming a direct shift in the thermal denaturation profile. The substitutions in the rod‐like extension domain of nsp8, that is, A21V and Q24R, increased the *T*_*M*_ by 3.9 and 5.7°C, respectively. M129I and T141M, located within the head domain, enhanced the stability to a greater extent. Notably, T141M had the most significant effect, increasing the *T*_*M*_ by 13.4°C. Concerning the nsp12 variants, the melting temperature of nsp12 P323L mutant was lower compared to the nsp12 wt; however, its aggregation temperature was proportionally higher (Figure 3d). This indicates that the P323L variant of nsp12 undergoes a faster denaturation that starts at a higher temperature. For a larger multidomain protein like nsp12, such a shift in thermal denaturation profile typically indicates more specific denaturation. Based on these data, we concluded that the P323L substitution had a stabilizing effect.

It has been shown that nsp7 and nsp8 also form a stable heterodimeric complex, which can further assemble into larger structures, such as tetramers (Biswal et al., 2021; Zhang et al., 2021), or even hexadecamers, as shown for SARS‐CoV (Zhai et al., 2005). We prepared combinations of nsp7 and nsp8 wt and mutant protein complexes in a 1:1 molar ratio, where each combination included one wt and one mutant protein, and assessed their thermal stability (Figure 3e). No significant shift of melting temperature was observed for the complexes of wt nsp8 with destabilizing nsp7 variants whereas for nsp8 mutant variants with wt nsp7, the thermal shift was similar to individual nsp8 subunits (Figure 3c,e).

To determine the effect of selected mutations on the stability of minimal RdRp complex, we combined wt nsp12 with each nsp7 individually, and then also with each variant of nsp8 mutant variants, as well as P323L nsp12 with wt nsp7 and wt nsp8 in a 1:1:2 ratio (Figure 3f). No significant shifts in *T*_*M*_ and *T*_*agg*_ were observed, that could be attributed to substitutions in either nsp7 or nsp8. Intriguingly, the substantial effect in individual nsp8 subunits and their complexes with nsp7 was not observed upon mixing with nsp12. This implies that these shifts were primarily driven by intrinsic stabilizing interactions in nsp8, which were then disrupted when nsp7 and nsp8 were recruited toward nsp12. Moreover, due to the significant differences in molecular weights between nsp7, nsp8, and nsp12, it is highly probable that nsp12 mainly contributes to the overall signal of the mixture. This is further supported by the observation that the same stabilizing effect was also evident when individual nsp12 P323L was combined with wt nsp7, and wt nsp8 in mixtures (Figure 3d,f).

### 2.3 Effect of mutations on protein–protein interactions and RdRp complex formation

The results of thermal stability experiments indicated that the selected amino acid substitutions do not significantly disrupt protein–protein interactions within the RdRp complex. Nonetheless, proper complex formation is extremely important as nsp12 gains most of its activity only upon binding to nsp7 and nsp8 (Peng et al., 2020). Therefore, we analyzed the impact of substitutions on RdRp complex formation in more detail.

**FIGURE 4:**
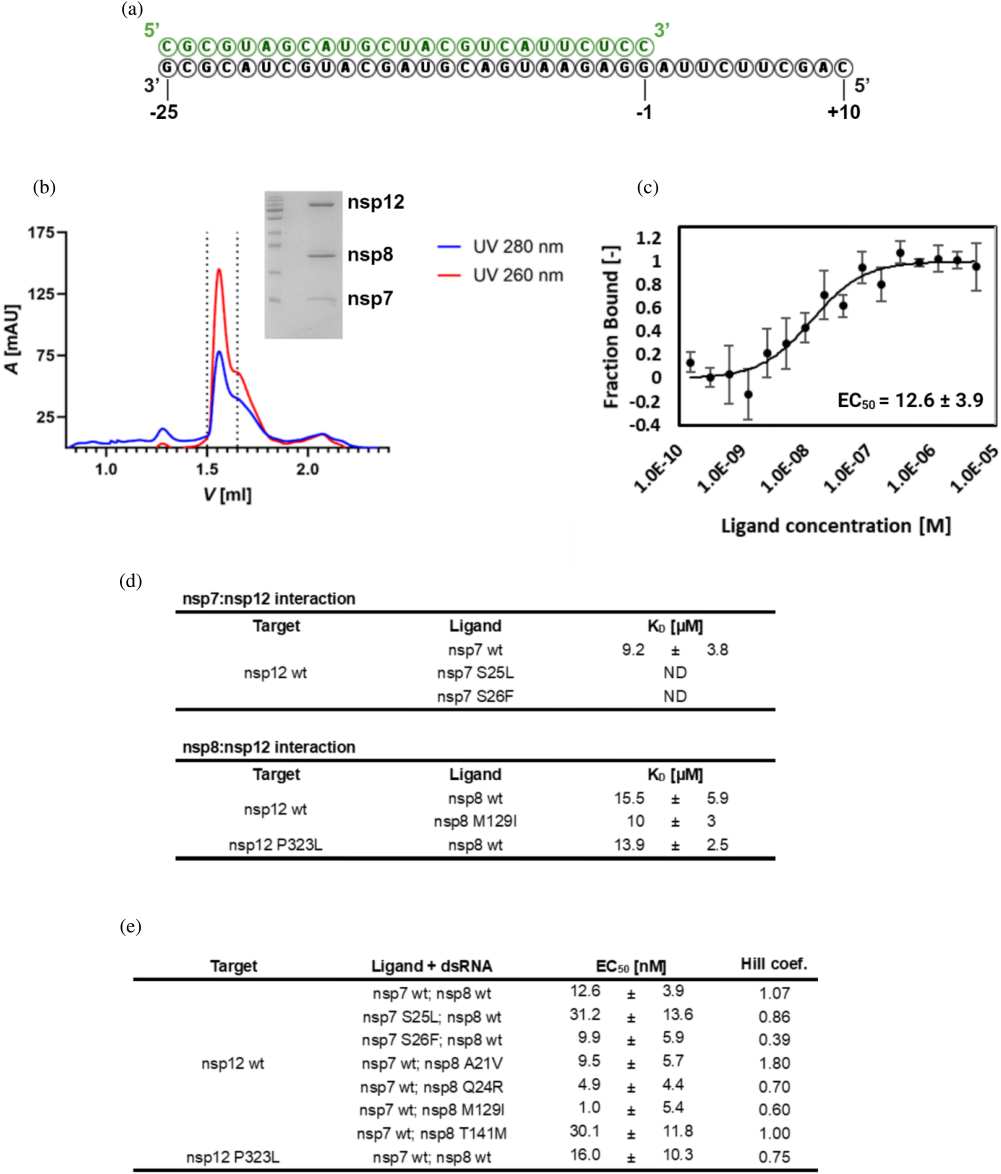
Verification of wild‐type RdRp assembly with dsRNA, protein–protein interactions, and RdRp assembly parameters. (a) dsRNA used for the analysis of dsRNA‐RdRp complex formation. (b) Analytical size exclusion chromatography of a preformed dsRNA‐RdRp complex. The complex was formed by combining individual wt components of the minimal RdRp complex, nsp7, nsp8, and nsp12 in a 1:2:2 molar ratio with the dsRNA (a) in a molar ratio of 1:1 (nsp12:dsRNA). Proteins were detected at 280 nm (blue) and RNA at 260 nm (red). The peak fraction was additionally evaluated by SDS‐PAGE (inlet). (c) MST analysis of the dsRNA‐RdRp complex. A subcomplex consisting of wt nsp7, wt nsp8 and dsRNA (a) in a 1:2:1 molar ratio (nsp7:nsp8:dsRNA) was used as a ligand for the wt nsp12 target. The Hill model for dose–response curve fitting was utilized to determine EC_50_. (d) Determined dissociation constants (K_D_) for nsp7:nsp12 and nsp8:nsp12 interactions by MST. (e) Experimental parameters of the assembly of dsRNA‐RdRp complexes with different subunit variants determined by MST with Hill model for dose–response curve fitting.

First, we tested the ability of the prepared wt proteins to form a complete dsRNA‐RdRp complex using an analytical size exclusion chromatography (SEC) as described by Hillen et al. (2020). The minimal RdRp complex was formed by combining wt nsp7, nsp8, and nsp12 in a 1:2:2 molar ratio, along with a short double‐stranded RNA with a 10 nucleotide (nt) overhang (Figure 4a) in a 1:1 molar ratio (dsRNA:nsp12). The proteins were detected spectrophotometrically at 280 nm and RNA at 260 nm (Figure 4b). In addition, the peak fraction was analyzed by SDS‐PAGE (Figure 4b, inlet) confirming the presence of a complex comprising all three components.

By using a microscale thermophoresis (MST) assay, we further investigated and quantified the impact of amino acid substitutions in the RdRp components on their mutual interactions and minimal dsRNA‐RdRp complex formation. Initially, we analyzed the binding affinity of nsp12 to both nsp7 and nsp8 (Figure 4d). The dissociation constant (K_D_) for wt nsp12 and wt nsp7 was about 9 μM. However, the K_D_ for the S25L and S26F could not be determined under the same experimental conditions due to insufficient working concentration of nsp7 caused by precipitation. The K_D_ reflecting the interaction between nsp12 and nsp8 was determined to be in the micromolar range both for wt and mutants with substitutions at nsp12 and nsp8 interface, specifically, nsp8 M129I and nsp12 P323L. Notably, neither of the two analyzed substitutions significantly affected the interaction (Figure 4d).

To evaluate the impact of amino acid substitutions on the assembly and stability of the whole minimal RdRp complex with dsRNA, we used a protocol published by Kim et al. (2023). The subcomplex assembled by combining nsp7, nsp8, and RNA in a molar ratio 1:2:1, was used as a ligand with nsp12 serving as a target molecule in the MST assay (Figure 4c). In these MST experiments, we used the same dsRNA as employed in the aforementioned SEC analysis for the complex formation (Figure 4a). Instead of K_D_ mode dose–response curve fitting, we employed the Hill model for multivalent interactions, which provides information about the cooperativity of the interaction and the half‐maximal effective concentration (EC_50_), thus representing the concentration at which 50% of the labeled nsp12 binds to ligands (Seidel et al., 2013). The EC_50_ of the RdRp wt complex was in the nanomolar range (Figure 4c), in contrast to the micromolar range K_D_ observed for nsp7‐nsp12 or nsp8‐nsp12 interactions (Figure 4). We next investigated the capability of nsp7, nsp8, or nsp12 variants to form the minimal RdRp complex and quantified the interaction parameters for each mutant. The alterations in complex formation characterized by EC_50_ were predominantly negligible (Figure 4e). However, the increased EC_50_ observed for the S25L substitution in nsp7 and T141M in nsp8, suggests their negative effect on complex formation. Moreover, except for nsp8 A21V, all mutant variants exhibited decreased Hill coefficient, indicating negatively affected cooperativity during the assembly.

### 2.4 Mutations influence the overall RdRp activity

**FIGURE 5:**
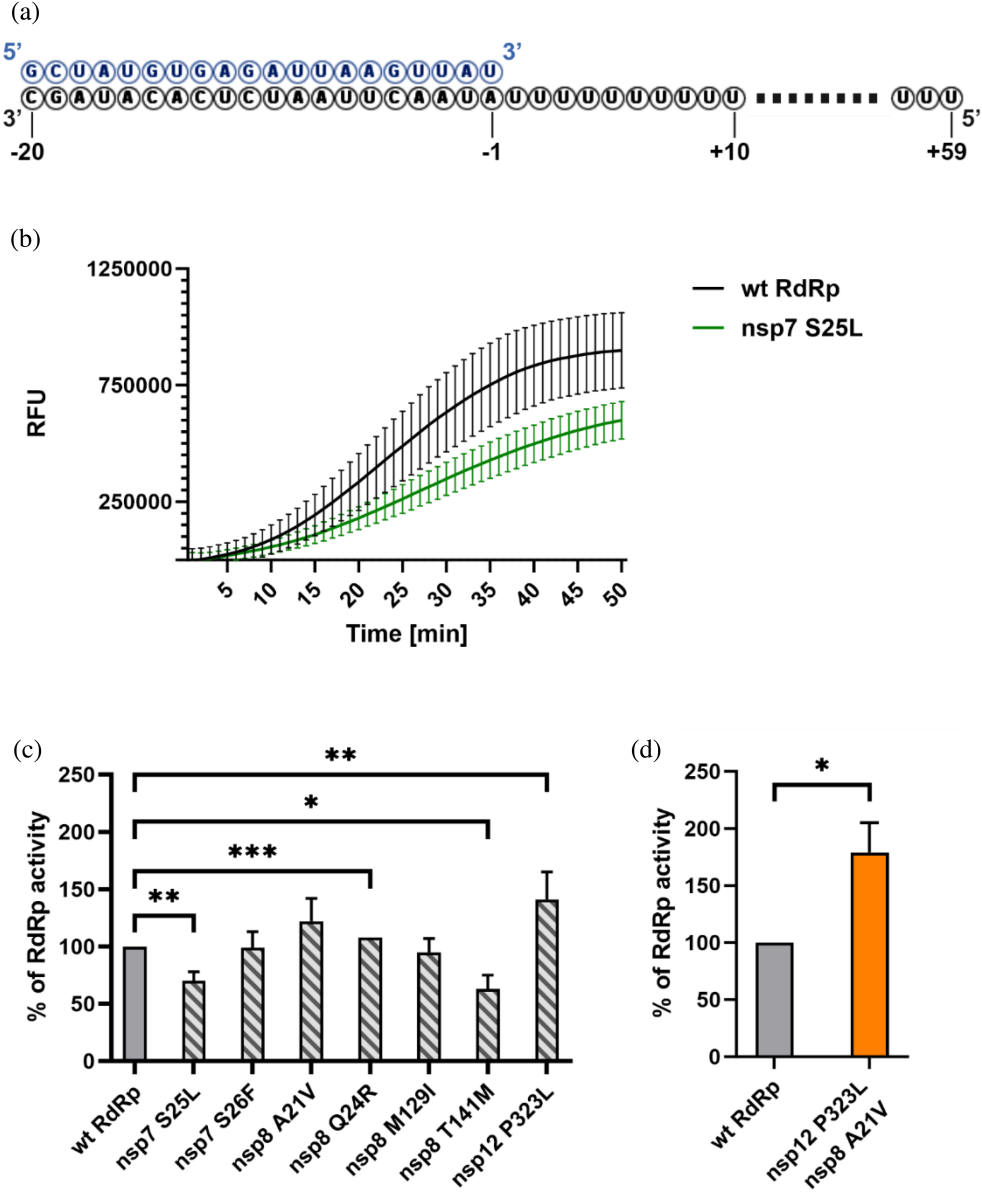
Amino acid substitutions of individual RdRp proteins alter the polymerase activity. (a) A Primed polyU RNA template used to analyze the RdRp activity. (b) Comparison of the fluorescence curves for RdRp complex of wt nsp7, wt nsp8 and wt nsp12 (labeled “wt RdRp”) and nsp7 S25L, wt nsp8 and wt nsp12 (labeled “nsp7 S25L”). (c) RNA polymerase activity of different RdRp variants compared with a combination of wt nsp7, wt nsp8, and nsp12. Each variant comprised one subunit with an amino acid substitution, for example, nsp7 S25L represents a combination of nsp7 S25L, wt nsp8, and wt nsp12. (d) The most significant increase in RdRp activity was observed for a complex of wt nsp7, nsp8 A21V, and nsp12 P323L. Results represent statistically processed data from at least three independent measurements. Outliers were excluded using a Q‐test at a 90% significance level. A paired two‐tailed *t* test was utilized to compare results with wt RdRp with indicated *p*‐values (*p ≤ 0.05; **p ≤ 0.01; ***p ≤ 0.001). The percentage of RdRp activity was calculated by comparing the area under the curve (AUC) with the AUC of wt RdRp. RFU—relative fluorescence units.

As the protein stability of all three RdRp subunits and complex formation were influenced by the amino acid substitutions, we hypothesized that the overall enzymatic activity of the RdRp complex might be affected as well. To test this hypothesis, we adapted a fluorometric RNA extension assay used for the analysis of ZIKA virus polymerase activity (Sáez‐Alvarez et al., 2019), with adjustments for the SARS‐CoV‐2 RdRp (Jones et al., 2022). Briefly, the activity assay utilizes a 20 nt RNA primer RNA annealed to a 79 nt template RNA, which includes a complementary region and a 59 nt polyU 5′ overhang (Figure 5a). Elongation of the primer RNA was measured using SYBR® Green I, an intercalating fluorescent dye. Fluorescence was measured in real‐time generating a fluorescence curve for each reaction. To compare the enzymatic activities of the protein variants, the area under the curve (AUC) was calculated for each fluorescence curve, with representative examples shown in Figure 5b. The AUC of a reaction comprising wt nsp7, nsp8, and nsp12 served as a reference standard, set to 100%, and the results for the mutants are presented as a percentage of the wt RdRp activity. For each substitution, at least three independent measurements were conducted.

RdRp activity was initially measured using nsp7‐nsp8‐nsp12 complexes, each containing a single protein with an amino acid substitution. The S25L substitution in nsp7 negatively affected complex formation, leading to a decrease in RdRp activity to 70.2 ± 8.3%. In contrast, the S26F substitution in nsp7 did not significantly affect the enzymatic activity. The impact of substitutions in nsp8 on RdRp activity varied depending on the location of the respective mutated residue. Substitutions within rod‐like extensions of nsp8 slightly activated the RdRp, specifically, Q24R increased the activity by 7.8 ± 0.3%. Conversely, the M129I substitution in the head domain of nsp8 had a negligible effect on RNA polymerase activity, and the exchange of T141 per methionine decreased the activity to 63.1 ± 12.1% compared to the wt RdRp (Figure 5c).

The P323L variant of nsp12 was previously proposed to enhance the RNA polymerase activity (Kim et al., 2023). Using the fluorometric method, we also observed an increase in the activity for the P323L nsp12 variant to 141.3 ± 24.0% relative to the wt nsp12 (Figure 5c). This particular nsp12 variant was first identified in February 2020 (Pachetti et al., 2020) and during the pandemic, was found to co‐occur with nearly every substitution in nsp7 and nsp8, except for M129I in nsp8 (covidcg.org). Therefore, we also evaluated the relative activity of RdRp complexes consisting of the nsp12 P323L variant in combination with various nsp7 and nsp8 variants. A notable synergistic effect was observed especially between nsp12 P323L and nsp8 A21V mutants (Figure 5d), which elevated the overall RdRp activity to 178.9 ± 25.7% compared to the wt RdRp complex.

## 3 DISCUSSION

Following the emergence of COVID‐19, the extensive transmission of SARS‐CoV‐2 led to the development of numerous variants carrying mutations which resulted in amino acid substitutions also identified in the proteins constituting the SARS‐CoV‐2 RdRp complex (Figure 1) potentially influencing virulence or resistance. In this study, we selected representative mutations in RdRp subunits, specifically S25L and S26F substitutions in nsp7; A21V, Q24R, M129I, and T141M in nsp8 and a dominant substitution P323L in nsp12. Corresponding proteins were cloned, expressed, and purified for a multiscale characterization of the phenotypic impact of the mutations.

The substitutions within all three subunits of the SARS‐CoV‐2 RdRp complex significantly influenced their thermal stability. The scale of thermal shift observed in both nsp7 and nsp8 mutants (Figure 3b,c) suggests that the effect of the substitutions is not solely intrinsic, but most likely due to the changes in the interactions ultimately leading to the formation of the RdRp complex. Unfortunately, reports on nsp7 self‐interactions have been diverse (Courouble et al., 2021; Wilamowski et al., 2021), making it challenging to determine whether the effect is due to a reduced multimerization. On the contrary, nsp8 was shown to form homodimers and homotetramers in solution (Wilamowski et al., 2021; Xu, Jiang, et al., 2022), indicating that the thermal shift could be induced as a result of enhanced multimerization. Regarding the nsp7:nsp8 complexes, thermal stability was primarily affected by the nsp8 subunit variant (Figure 3c,e). This suggests that the effect of various substitutions might extend from nsp8 homomultimers to nsp7:nsp8 heteromultimers. Moreover, none of the analyzed amino acid substitutions is located near the nsp7:nsp8 interface (Biswal et al., 2021), implying that these substitutions should not interfere with the complex formation as observed for various nsp7 variants (Figure 3e). While the complex of nsp7 with the C‐terminal domain of nsp8 is structurally well characterized (Zhai et al., 2005), much less is known about the position of the N‐terminal extension within these complexes. Additionally, the structure of nsp8 homomultimers has not been determined yet. It is known that the N‐terminal extension may adopt multiple conformations, as shown for the SARS‐CoV nsp7:nsp8 hexadecamer (Xiao et al., 2012) or the Feline coronavirus nsp7:nsp8 heterotrimer (Wilamowski et al., 2021). In addition, Wilamowski et al. (2021), proposed possible interactions between N‐ and C‐ termini of nsp8 in solution, combining SAXS and SANS techniques. These interactions might be enhanced by selected substitutions in nsp8, potentially increasing the stability of both nsp8 homomultimers and nsp7:nsp8 complexes.

As indicated by the thermal unfolding assay, selected amino acid substitutions did not affect the RdRp complex formation (Figure 3f). To verify these findings, we quantified the protein–protein interactions using an MST assay. The interactions between nsp7:nsp12 and nsp8:nsp12 showed a K_D_ in micromolar range. In contrast, the formation of the RdRp complex with both nsp7 and nsp8 subunits along with dsRNA occurred within the nanomolar EC_50_. This suggests that the presence of all RdRp subunits together with the dsRNA greatly enhances the individual interactions that lead to the formation of the dsRNA‐RdRp complex, which is consistent with previously published data by Kim et al. (2023). However, our results slightly differed from that of Kim et al. (2023). First, the EC_50_ in our study indicated greater affinity during the assembly of wt RdRp and second, we found no significant difference in complex formation between wt and P323L nsp12 variants. These discrepancies could be attributed to the differences in the nsp12 purification protocol. The protocol used in this study consisted of multiple steps including tag cleavage, while Kim et al. (2023) utilized nsp12 purified with a single‐step NiNTA chromatography.

Our results showed that even a single amino acid substitution in any of the protein subunits of SARS‐CoV‐2 RdRp could alter the enzymatic activity of the complex (Figure 5c). Noteworthy, substitutions within nsp7 and nsp8 did not change the RdRp activity as substantially as P323L in nsp12, and even in comparison with other studied mutations in nsp12 (Miropolskaya et al., 2023), the impact was less significant. As anticipated, the differences in enzymatic activity align with alterations in the biophysical properties of the proteins. For instance, S25L in nsp7 and T141M in nsp8 showed higher EC_50_ values in MST experiments, subsequently exhibiting a notable decrease in RdRp activity. Conversely, nsp7 S26F and nsp8 M129I, which largely retained the relative RdRp activity, exhibited comparable EC_50_ values to their respective wt variants. Intriguingly, both of these mutations decreased the Hill coefficient of the complex assembly, suggesting that the cooperativity of the assembly is unrelated to the overall enzymatic activity. Concerning the mutations in the N‐terminal rod‐like extension domain of nsp8, both A21V and Q24R mutants tended to increase the catalytic activity of the RdRp; however, with varying effects on assembly parameters. This increase in activity does not seem to depend on the enhanced ability of complex formation. Instead, it is plausible that these substitutions promote intermolecular interactions within the helix‐turn‐helix motif at the N‐terminus (Figure 2c) (Hillen et al., 2020; Treviño et al., 2023), as suggested by a slight increase in thermal stability observed for these nsp8 variants (Figure 3c,e). This finding is similar to the increase in activity observed by Subissi et al. (2014) for SARS‐CoV nsp8 mutant with S11A substitution located within this motif. Moreover, some of our experimental results differ from the in silico analyses of amino acid substitutions in subunits of SARS‐CoV‐2 RdRp. For example, Reshamwala et al. (2021) predicted that M129I in nsp8 and S26F in nsp7 might potentially benefit the RdRp complex by increasing the surface complementarity of the subunits. However, we observed that S26F and M129I had no significant effect on either complex formation or catalytic activity. In contrast, T141M substitution in nsp8 was predicted to destabilize the RdRp complex by Senthilazhagan et al. (2023), which was confirmed by our data. Both mentioned studies suggested that substituting S25 in nsp7 per leucine could stabilize the RdRp, but our results showed the opposite (see Figures 4e and 5c). Finally, while the P323L substitution in nsp12 is regarded as a stabilizing mutation, our data revealed that although it stabilized the protein itself (Figure 3d,e), it did not enhance the protein–protein interactions (Figure 4d,e).

A comparison of the overall nature of the analyzed mutant RdRp variants analyzed in this study allows us to conclude that certain mutations have a distinct impact on RNA replication. In particular, mutations in the nsp8 extensions, specifically A21V and Q24R, and P323L in nsp12, have been demonstrated to be beneficial for RNA replication. Conversely, mutations such as S25L in nsp7 and T141M in nsp8 have been demonstrated to have a generally negative impact, potentially decreasing viral fitness. However, it is important to emphasize the co‐occurrence of these mutations, particularly in connection with nsp12 P323L. Both negative mutations (S25L and T141M) were found primarily to co‐occur with the P323L variant of nsp12 suggesting that the negative effects might be compensated by the beneficial impact of P323L. On the other hand, the co‐occurrence of nsp12 P323L with either nsp8 A21V or nsp8 Q24R could potentially result in the emergence of even more pathogenic SARS‐CoV‐2 variants. The favorable character of P323L substitution in nsp12 is in agreement with previous research indicating that SARS‐CoV‐2 variants carrying this mutation in RdRp exhibit higher infectivity and enhanced selective advantage (Goldswain et al., 2023; Kannan et al., 2020; Kim et al., 2023). Additionally, this mutation was also linked to an increased mutation rate during RNA replication, potentially accelerating virus evolution (Pachetti et al., 2020). According to sequence analysis (covidcg.org), the only mutation that would not co‐occur with nsp12 P323L is the M129 I substitution in nsp8 M129I. This mutation might have represented an alternate evolutionary pathway for the virus, which was suppressed by the nsp12 P323L variants with enhanced RNA replication. Other mutations in nsp7 and nsp8 were identified in a wide range of isolates without any apparent mutual relation or phenotypic effect, suggesting their evolution was presumably random. Notably, among these mutations, only the nsp7 S25L variant, which negatively impacted RdRp function, has been linked to a specific locally predominant SARS‐CoV‐2 lineage—the B.1.497 subvariant—circulating in South Korea in 2020 (Kwon et al., 2021; Singer et al., 2020). Furthermore, considering the observed impacts on the thermal stability of individual RdRp subunits, it is also noteworthy that there might be potential implications for the course of COVID‐19. According to Kim et al. (2023), the nsp12 P323L mutation affects the RdRp activity at different temperatures, directing the virus to replicate more rapidly in the upper respiratory tract. Similar effects might thus result from the analyzed mutations in this study.

Mutations in RdRp subunits may also represent an obstacle to the design of non‐nucleoside or allosteric RdRp inhibitors. Several strategies for such inhibitors have been proposed. For example, in the study of Barakat et al. (2022) they identified three potential binding pockets for such inhibitors in nsp12. The most druggable pocket was identified in the interface domain of nsp12. The proline at position 323 within this pocket appears to be particularly important due to the structural changes induced by the P323L substitution (see Figure 2f). This effect was further explored in more detail in a computational study by Mutlu et al. (2022) focusing on inhibitors of the nsp8a:nsp12 interaction interface. They proposed that the P323L mutation alters local structural motifs, thereby changing the binding properties of the pocket for certain molecules. It is also notable that the efficacy of the inhibitor binding between the nsp8a:nsp12 interface may be influenced by mutant variants of nsp8 with a stronger affinity for nsp12. The interaction interfaces of nsp12 with both nsp8a and nsp7 were also highlighted as a potential inhibitor binding site by Ruan et al. (2021) based on efficient docking of several small molecules in these regions. An interesting strategy utilizing peptide inhibitors of nsp8a:nsp12 and nsp7:nsp12 interactions was published by Jena and Pant (2023). This strategy is however directly related to mutations affecting these interactions. The efficacy of peptides competing with nsp7 or nsp8 for the binding site would presumably vary with the dynamics of RdRp complex assembly.

In conclusion, our findings elucidate the impact of naturally occurring amino acid substitutions within the RdRp proteins of SARS‐CoV‐2. Understanding these changes is crucial, as they can significantly influence the interaction dynamics of the RdRp complex and the enzymatic activity. Considering the importance of RdRp in the replication cycle of the virus, our results are particularly important for the development of new antiviral strategies. In particular, mutations impacting the assembly of RNA‐RdRp complex should be taken into consideration to ensure sufficient efficacy of newly developed drugs.

## 4 METHODS

### 4.1 Preparation of expression vectors

Expression vectors encoding 6 × His‐TEV‐nsp7 (#154757), 6 × His‐TEV‐nsp8 (#154758) and 6 × HisMBP‐TEV‐nsp12 (#154759) were obtained from AddGene (Hillen et al., 2020). Efficient mutagenesis independent of ligation (EMILI) (Füzik et al., 2014) was utilized to introduce mutations in the respective sequences. Briefly, primers encoding the mismatch mutations (Table S1) were used in an inverse PCR to amplify the entire expression vector. Subsequently, the methylated template DNA was digested with DpnI for 90 min at 37°C. The mixtures were then incubated with T4 DNA polymerase for 5 min at 22°C to generate sticky ends. To inactivate T4 DNA polymerase, EDTA was added to the final concentration of 2.5 mM, and the temperature was increased to 72°C for 20 min. The whole reaction mixture was then used for the transformation of *E. coli* DH5α competent cells. Finally, selected colonies were used to amplify the vector with introduced mutations. Each expression vector was verified by Sanger sequencing.

### 4.2 Protein production and purification

#### 4.2.1 nsp7 and nsp8

6 × His‐TEV‐nsp7 and 6 × His‐TEV‐nsp8 variants were separately expressed in *E. coli* BL21 DE3 CodonPlus RIL. The cells were grown in LB medium at 37°C to OD_600_ 0.6. Subsequently, the cultures were cooled to 18°C, induced with IPTG at a final concentration of 0.4 mM and protein expression continued for 16 hours at 18°C. Cells were then separated from the media by centrifugation (3000 × g, 15 min), resuspended in nsp7/8 Lysis buffer (50 mM Na‐HEPES pH 7.4, 300 mM NaCl, 30 mM imidazole, 10% glycerol, 2 mM β‐mercaptoethanol) and disrupted by high pressure using a One Shot Cell Disruptor (Constant Systems) at 1.9 kbar. In the case of nsp7, Triton X‐100 was added before the disruption to the final concentration of 0.15%. The lysate was then cleared by centrifugation (50,000 × g, 30 min), and the supernatant was loaded on HisTrap™ HP (Cytiva) preequilibrated in Lysis buffer. The column was washed with High Salt buffer and Low Salt buffer containing 1000 mM NaCl and 150 mM NaCl, respectively. The proteins were then eluted with Elution buffer (50 mM Na‐HEPES pH 7.4, 150 mM NaCl, 500 mM imidazole, 10% glycerol, 2 mM β‐mercaptoethanol). Peak fractions were collected, and His‐tagged TEV protease was added at a 1:10 w:w ratio. The mixture was dialyzed against 3 L of Low salt buffer at 4°C for 16 h to remove imidazole. After dialysis, the sample was loaded on the HisTrap™ HP column equilibrated in Low Salt buffer to remove the uncleaved protein and His‐TEV protease. Flowthrough fraction containing the cleaved protein was collected and dialyzed against 3 L of Nsp7/8 Storage Buffer (20 mM Na‐HEPES pH 7.4, 150 mM NaCl, 5% glycerol, 1 mM TCEP). Finally, the samples were concentrated, aliquoted, and stored at −80°C until use.

#### 4.2.2 nsp12

Bacmids containing 6 × His‐MBP‐TEV‐nsp12 variants were generated using the Bac‐to‐Bac™ baculovirus expression system (Invitrogen). *Sf9* cells were transfected with the bacmid DNA and baculovirus‐containing cell supernatants were harvested 5 days after transfection. The resulting baculoviral stock was amplified to achieve optimal infectivity. Subsequently, a suspension of *Sf9* cells (3 × 10^6^ cells/ml) was infected with the baculoviral stock in a 1:100 ratio. The expression of the 6 × His‐MBP‐TEV‐Nsp12 protein was carried out for 96 h after which the cells were harvested by centrifugation (3000 × g, 15 min). To isolate the protein, the cell pellet was resuspended in Lysis buffer (50 mM Na‐HEPES pH 7.4, 300 mM NaCl, 30 mM imidazole, 3 mM MgCl_2_ 10% glycerol, 5 mM β‐mercaptoethanol) and homogenized utilizing One Shot Cell Disruptor (Constant Systems) operating at 1.0 kbar. Cell lysate was subsequently clarified by centrifugation at 70,000 × g for 30 min and ultracentrifugation at 235,000 × g for 60 min. The obtained supernatant was then loaded on HisTrap™ HP (Cytiva) in Lysis buffer and washed with High salt buffer containing 1000 mM NaCl. A MBPTrap™ HP (Cytiva) column was connected downstream to the HisTrap™ HP, from which the protein was then eluted with 500 mM imidazole. HisTrap™ column was subsequently disconnected and the MBPTrap™ HP was washed with additional five column volumes of Lysis buffer. Finally, nsp12 was eluted with a buffer containing 116.9 mM maltose. 6 × His‐TEV protease was added to the eluate in a 1:10 w:w ratio and the sample was dialyzed against 3 L of Lysis buffer at 4°C for 16 h. Reverse IMAC was utilized to remove TEV protease, cleaved purification tags, and uncleaved protein. Nsp12 was further purified using heparin affinity chromatography. For this purpose, the sample was diluted to 80 mM NaCl using a Dilution buffer (20 mM Na‐HEPES pH 7.4, 1 mM MgCl_2_, 10% glycerol, 5 mM β‐mercaptoethanol) and loaded on HiTrap heparin HP (Cytiva) in Binding buffer (20 mM Na‐HEPES pH 7.4, 80 mM NaCl, 1 mM MgCl_2_, 10% glycerol, 5 mM β‐mercaptoethanol). The protein was then eluted with a linear gradient of NaCl from 80 to 1000 mM. The fractions containing nsp12 were pooled and dialyzed for 16 h against 3 L of Storage buffer (20 mM Na‐HEPES pH 7.4, 300 mM NaCl, 1 mM MgCl_2_, 10% glycerol, 1 mM TCEP). The final sample was concentrated and frozen at −80°C as single‐use aliquots.

### 4.3 Analytical size exclusion chromatography

RNA 25‐mer 5′‐CGCGUAGCAUGCUACGUCAUUCUCC‐3′ and 35‐mer 5′‐CAGCUUCUUAGGAGAAUGACGUAGCAUGCUACGCG‐3′ were first mixed in a 1:1 ratio in Annealing buffer (20 mM Na‐HEPES pH 8, 10 mM KCl, 6 mM MgCl_2_, 0.01%Triton X‐100, 1 mM DTT). The mixture was then heated at 95°C for 5 min and stepwise cooled to room temperature to generate an RNA duplex (Assembly RNA). Wild‐type nsp12 was subsequently added to the RNA in a 1:1 molar ratio whereas nsp7 and nsp8 were both supplemented in a 2:1 molar ratio. The complete mixture was incubated at 4°C for 2 h to allow for stable assembly. Subsequently, the RNA‐RdRp complex was loaded on Superdex Increase 3.2/300 column (Cytiva) in RdRp Assembly buffer (20 mM Na‐HEPES pH 7.4, 100 mM NaCl, 1 mM MgCl_2_, 1 mM TCEP). UV absorbance at 260 and 280 nm was monitored throughout the analysis.

### 4.4 Thermal unfolding assay

Nsp7, nsp8, and nsp12 variants were first diluted to a suitable concentration to facilitate the fluorescence signal − 50 μM for both nsp8 and nsp7 and 10 μM for nsp12. To analyze the nsp7:nsp8 dimers and nsp7:nsp8:nsp12 mixtures, the proteins were mixed in 1:1, or 1:2:1 ratio, respectively. Thermal unfolding parameters were then determined using a combination of nano‐differential scanning fluorimetry (nanoDSF) and turbidimetry using Prometheus Panta (Nanotemper). Protein solutions were heated at 2°C per minute from 25 to 95°C. Intrinsic fluorescence (λ_ex_ = 280 nm, λ_em_ = 330 and 350 nm) and turbidity were measured throughout the process. Results were analyzed using the Panta Analysis software (Nanotemper). Melting temperature *T*_*M*_ was collected as the inflection point of the 330/350 fluorescence ratio curve whereas *T*_*Agg*_ represents the onset temperature of turbidity increase.

### 4.5 Microscale thermophoresis assay

Wt and P323L variants of nsp12 were labeled with NHS‐fluorescent labels utilizing the Protein labeling kit (Nanotemper). Labeled proteins were then diluted to either 105 nM for protein–protein interactions or 52.5 nM for complex formation and utilized as a target molecule. The stock solutions of nsp7 or nsp8 variants were used as a ligand to analyze the protein–protein interactions. To evaluate complex formation, the ligand was prepared by mixing various variants of nsp7 and nsp8 with Assembly RNA in a 1:2:1 ratio (nsp7:nsp8:RNA) to the final concentrations of 10, 20, and 10 μM. Every nsp7:nsp8:RNA subcomplex was incubated for 15 min at room temperature before the next step. The respective ligands were then diluted to make 16 twofold dilutions. For the experiment, 10 μL of each ligand dilution was mixed with 10 μL of the respective target molecule and the obtained mixtures were incubated for an additional 15 min and subsequently cleared by centrifugation. Each mixture was then loaded into Monolith NT.115 capillaries (Nanotemper). The measurements were conducted using the Monolith NT.115 instrument (Nanotemper) at 25°C with the setting adjusted to 20% LED power and medium MST power. Three independent measurements were carried out for each binding experiment. The obtained data were analyzed using MO affinity (Nanotemper) using MST‐on time 19 to 20 s for protein–protein interactions and 0.5 to 1.5 s for complex formation. K_D_ mode fitting was utilized to evaluate protein–protein interactions whereas complex formation was assessed through the Hill model for multivalent binding, resulting in the determination of EC_50_ and Hill coefficient values.

### 4.6 Fluorometric RNA extension assay

The Substrate RNA for the extension assay was prepared as follows: The RNA template 5′‐U_59_AUAACUUAAUCUCACAUAGC‐3′ and RNA primer 5′‐GCUAUGUGAGAUUAAGUUAU‐3′ were mixed in 1:1 molar ratio in Annealing buffer (20 mM Na‐HEPES pH 8, 10 mM KCl, 6 mM MgCl_2_, 0.01%Triton X‐100, 1 mM DTT). The RdRp activity was subsequently monitored in real‐time using SYBR® Green I, which causes an increase in emitted fluorescence through binding exclusively to double‐stranded nucleic acid. The reaction mixtures were prepared by mixing nsp7, nsp8, and nsp12 in a 1:3:3 molar ratio with final concentrations of 0.63, 1.88, and 1.88 μM along with 250 μM rATP and 5 μM SYBR® Green I in Reaction buffer (20 mM Na‐HEPES pH 8, 10 mM KCl, 6 mM MgCl_2_, 0.01%Triton X‐100, 1 mM DTT). The mixtures were then transferred to a 96‐well plate and reactions were initiated by adding Substrate RNA to the final concentration 0.5 μM. The well‐plate was subsequently incubated at 30°C for 50 min in the thermocycler QuantStudio 5 real‐time PCR system (Applied Biosystems). SYBR® Green I fluorescence was recorded each minute throughout the incubation, resulting in a fluorescence curve for each reaction.

### 4.7 RdRp activity evaluation

To evaluate the activity of the RdRp, the area under the curve (AUC) was calculated as a numerical integration of each obtained fluorescence curve using the midpoint method. Therefore, the AUC represents a sum of rectangles, where the area of each rectangle is calculated by multiplying the average of two adjacent values for fluorescence signal by a step of 1 min. The calculated AUC for each variant was referred to AUC of the wild‐type RdRp reaction. Data were collected from at least three independent measurements with two duplicate reactions within each experiment. Outliers were excluded by using the Q‐test. A two‐tailed paired *t* test was utilized to statistically compare results with the respective references.

## AUTHOR CONTRIBUTIONS

**Matěj Danda:** Methodology; validation; writing – original draft; investigation; data curation. **Anna Klimešová:** Methodology; data curation. **Klára Kušková:** Methodology; data curation. **Alžběta Dostálková:** Methodology; data curation; supervision. **Aneta Pagáčová:** Methodology; data curation. **Jan Prchal:** Methodology; data curation; validation. **Marina Kapisheva:** Methodology; validation; visualization; software. **Tomáš Ruml:** Writing – review and editing; supervision. **Michaela Rumlová:** Conceptualization; funding acquisition; writing – review and editing; project administration; formal analysis; resources; supervision.

## CONFLICT OF INTEREST STATEMENT

The authors declare no competing interests.

## Supporting information


**Data S1.** Figure S1: Example Thermal unfolding profiles. Table S1: List of oligonucleotides used for mutagenesis.
