# Supplementary material for: Biochemical characterization of naturally occurring mutations in SARS‐CoV‐2 RNA‐dependent RNA polymerase
Source: Protein Sci. 2024 Aug 15;33(9):e5103. doi: 10.1002/pro.5103 (PMC11325161; doi:10.1002/pro.5103)
Supplement: Supplementary file 1 — Data S1. Figure S1: Example Thermal unfolding profiles. Table S1: List of oligonucleotides used for mutagenesis. [file PRO-33-e5103-s001.pdf]

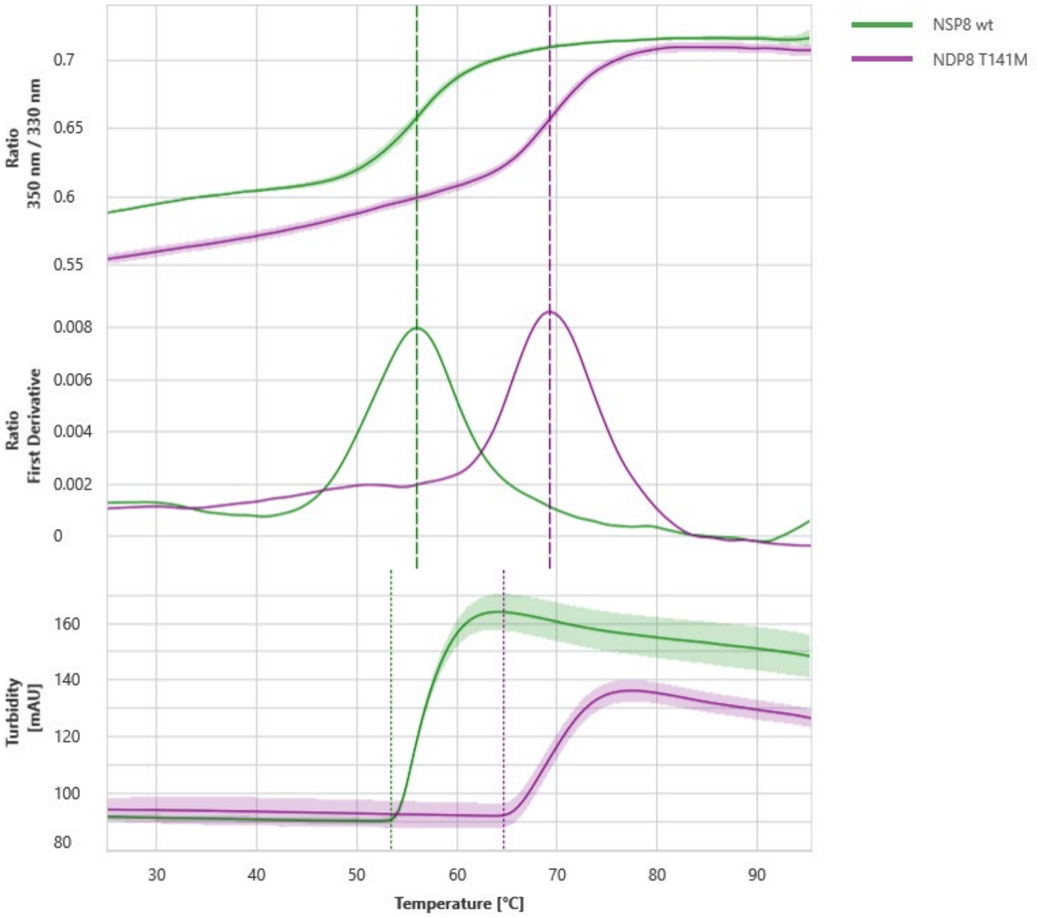

Figure S1: Example Thermal unfolding profiles. Thermal unfolding of nsp8 wt (green) and nsp8 T141M (violet). Ratio of fluorescence at 350 nm and 330 nm (top) and first derivative of the ratio (middle) were used to collect melting temperatures (T_M) indicated by dashed lines. Turbidimetric measurement (bottom) was utilized to obtain onset aggregation temperature (T_{Agg}) shown as dotted lines.

Table S1: List of oligonucleotides utilized for mutagenesis

Oligonucleotide	Sequence
nsp12 P323L fwd	GTGTTCCCTCTAACCTCCTTCGGTCCTCTCG
nsp12 P323L rev	CGAAGGAGGTTAGAGGGAACACAGTGG
nsp7 S25L fwd	GTTGAAAGCCTCAGCAAACTGTGGGCACAGTG
nsp7 S25L rev	CACAGTTTGCTGAGG CTT TCAACACGCAGCTG
nsp7 S26F fwd	GTTGAAAGCAGCTTCAAACTGTGGGCACAGTG
nsp7 S26F rev	CACAGTTTGAAGCTGCTTTCAACACGCAGCTG
nsp8 A21V fwd	CAAGAGGTATATGAACAGGCAGTTGCC
nsp8 A21V rev	CCTGTTCATATACCTCTTGTGCGGTTGC
nsp8 Q24R fwd	CTGCCCGTTCATATGCCTCTTGTGCGG
nsp8 Q24R rev	GGTGCCATCACACATGTTTTTATAGGTGTTGTAATC
nsp8 M129I fwd	GCAAAACTGATTGTGGTTATTCCGGAT
nsp8 M129I rev	GAATAACCACAATCAGTTTTGCTGCGGTG
nsp8 T141M fwd	CAACACCTATAAAAACATGTGTGATGGCACCACC
nsp8 T141M rev	GGTAAAGGTGGTGCCATCACACATGTTTTTATAGG
